# Establishment and Validation of CyberKnife Irradiation in a Syngeneic Glioblastoma Mouse Model

**DOI:** 10.3390/cancers13143416

**Published:** 2021-07-08

**Authors:** Claudius Jelgersma, Carolin Senger, Anne Kathrin Kluge, Anastasia Janas, Melina Nieminen-Kelhä, Irina Kremenetskaia, Susanne Mueller, Susan Brandenburg, Franziska Loebel, Ingeborg Tinhofer, Alfredo Conti, Volker Budach, Peter Vajkoczy, Gueliz Acker

**Affiliations:** 1 Department of Neurosurgery, Charité—Universitätsmedizin Berlin, Corporate Member of Freie Universität Berlin, Humboldt-Universität zu Berlin, and Berlin Institute of Health, Charitéplatz 1, 10117 Berlin, Germany; claudius.jelgersma@charite.de (C.J.); anastasia.janas@charite.de (A.J.); melina.nieminen@charite.de (M.N.-K.); irina.kremenetskaia@charite.de (I.K.); susan.brandenburg@charite.de (S.B.); franziska.loebel@charite.de (F.L.); alfredo.conti2@unibo.it (A.C.); peter.vajkoczy@charite.de (P.V.); 2 Department of Radiation Oncology, Charité—Universitätsmedizin Berlin, Corporate Member of Freie Universität Berlin, Humboldt-Universität zu Berlin, and Berlin Institute of Health, Augustenburger Platz 1, 13353 Berlin, Germany; carolin.senger@charite.de (C.S.); anne.kluge@charite.de (A.K.K.); ingeborg.tinhofer@charite.de (I.T.); volker.budach@charite.de (V.B.); 3 Charité CyberKnife Center, Augustenburger Platz 1, 13353 Berlin, Germany; 4 Department of Experimental Neurology and Center for Stroke Research, Charité—Universitätsmedizin Berlin, Corporate Member of Freie Universität Berlin, Humboldt-Universität zu Berlin, Berlin Institute of Health, Charitéplatz 1, 10117 Berlin, Germany; susanne.mueller1@charite.de; 5 NeuroCure Cluster of Excellence and Charité Core Facility 7T Experimental MRIs, Charité—Universitätsmedizin Berlin, Charitéplatz 1, 10117 Berlin, Germany; 6 IRCCS Istituto delle Scienze Neurologiche di Bologna, Via Altura 3, 40139 Bologna, BO, Italy; 7 Alma Mater Studiorum-Università di Bologna, Dipartimento di Scienze Biomediche e Neuromotorie (DIBINEM), Via Altura 3, 40139 Bologna, BO, Italy; 8 Berlin Institute of Health at Charité—Universitätsmedizin Berlin, BIH Academy, Clinician Scientist Program, Charitéplatz 1, 10117 Berlin, Germany

**Keywords:** CyberKnife, stereotactic radiosurgery, glioblastoma, GL261, mouse, radiobiology, small animal radiation

## Abstract

**Simple Summary:**

Stereotactic radiosurgery (SRS) provides precise high-dose irradiation of intracranial tumors. However, its radiobiological mechanisms are not fully understood. This study aims to establish CyberKnife SRS on an intracranial glioblastoma tumor mouse model and assesses the early radiobiological effects of radiosurgery. Following exposure to a single dose of 20 Gy, the tumor volume was evaluated using MRI scans, whereas cellular proliferation and apoptosis, tumor vasculature, and immune response were evaluated using immunofluorescence staining. The mean tumor volume was significantly reduced by approximately 75% after SRS. The precision of irradiation was verified by the detection of DNA damage consistent with the planned dose distribution. Our study provides a suitable mouse model for reproducible and effective irradiation and further investigation of radiobiological effects and combination therapies of intracranial tumors using CyberKnife.

**Abstract:**

CyberKnife stereotactic radiosurgery (CK-SRS) precisely delivers radiation to intracranial tumors. However, the underlying radiobiological mechanisms at high single doses are not yet fully understood. Here, we established and evaluated the early radiobiological effects of CK-SRS treatment at a single dose of 20 Gy after 15 days of tumor growth in a syngeneic glioblastoma-mouse model. Exact positioning was ensured using a custom-made, non-invasive, and trackable frame. One superimposed target volume for the CK-SRS planning was created from the fused tumor volumes obtained from MRIs prior to irradiation. Dose calculation and delivery were planned using a single-reference CT scan. Six days after irradiation, tumor volumes were measured using MRI scans, and radiobiological effects were assessed using immunofluorescence staining. We found that CK-SRS treatment reduced tumor volume by approximately 75%, impaired cell proliferation, diminished tumor vasculature, and increased immune response. The accuracy of the delivered dose was demonstrated by staining of DNA double-strand breaks in accordance with the planned dose distribution. Overall, we confirmed that our proposed setup enables the precise irradiation of intracranial tumors in mice using only one reference CT and superimposed MRI volumes. Thus, our proposed mouse model for reproducible CK-SRS can be used to investigate radiobiological effects and develop novel therapeutic approaches.

## 1. Introduction

Stereotactic radiosurgery with CyberKnife (CK-SRS), where high dose radiation is delivered from multiple angles adjusted in real time to the position of the irradiated subject, has increasingly been applied to treat intracranial tumors (e.g., glioblastoma (GBM) and brain metastases) with submillimeter precision [1,2,3]. Thus, CK-SRS with high single dose delivery is an alternative strategy to fractionated irradiation and serves as an additional treatment option for surgical approaches [4,5]. While GBM is the most common malignant brain tumor, brain metastases represent one of the major indicators of intracranial SRS application [6,7]. Because of the heterogeneous tumor biology and the associated development of resistance in monotherapy application, multimodal therapy approaches are a keystone to treat tumors synergistically from several directions [8]. In this regard, a combination of irradiation with chemo-, immune-, or targeted therapies offers promising therapeutic regimes [9].

However, despite the increasing clinical applications of single- or hypofractionated treatment, evidence-based radiobiological studies for the intracranial applications of high-dose radiation treatments are still lacking. Recent clinical studies on SRS have provided crucial insights into the safety and efficacy of hypofractionation with high single doses; however, the underlying radiobiological mechanisms remain poorly understood [10]. For instance, Ko et al. outlined the preclinical and clinical evidence on the differential radiobiological mechanisms underlying hypofractionated radiotherapy [10]. Overall, there is limited knowledge on the effects of radiosurgery on immune cells and their role in antitumor immunity. Furthermore, an increased risk of radionecrosis after high-dose SRS in comparison to fractioned radiotherapy has been reported and thus requires further investigation [11].

However, to focus on the radiobiology induced by CK-SRS in intracranial pathologies, a reliable in vivo model is required. Small animal studies on tumor treatment serve as a foundational basis for the development of new treatment strategies. Preclinical animal models may reflect the tumor microenvironment, especially if clinical treatment practice using SRS for orthotropic mouse models can be provided with radiation therapy conditions identical to those in humans. However, specialized image-guided small animal irradiation devices for preclinical research are only available in a few research facilities [12,13,14,15].

The effects of irradiation on healthy whole brain have been extensively investigated using mouse models [16,17,18,19,20,21,22,23,24,25,26,27,28,29,30]. Nevertheless, only a few studies on the irradiation of only one hemisphere of healthy brains have been conducted [31,32,33,34,35], and studies on the effects of irradiation on tumors in mouse models are rare [12,14,36,37,38,39]. To date, there are only three studies on the irradiation of a mouse model using dedicated radiosurgical linear accelerators, two of which were performed using Gamma Knife [34,39] whereas the other used CK [33]. Additionally, CK irradiation was technically established for irradiation of a healthy mouse brain hemisphere, but without addressing underlying radiobiological aspects [33]. Thus, there is an urgent need to establish preclinical models that closely mimic human tumors, which could facilitate the development novel therapeutic interventions that improve clinical management.

## 2. Materials and Methods

### 2.1. Animals

Twenty-one female C57BL/6N mice (Charles River Laboratories, Sulzfeld, Germany) at 13.5 ± 3.5 weeks old and with an average weight of 22.0 ± 1.2 g were used. They were kept in a 12-h light-dark cycle and were fed ad libitum. All animal experiments were conducted according to the German Law for Animal Protection, controlled by LAGeSo (Berlin, Germany) under the registration number G0221/17. ARRIVE guidelines were respected.

### 2.2. Tumor Cell Inoculation

**Figure 1:**
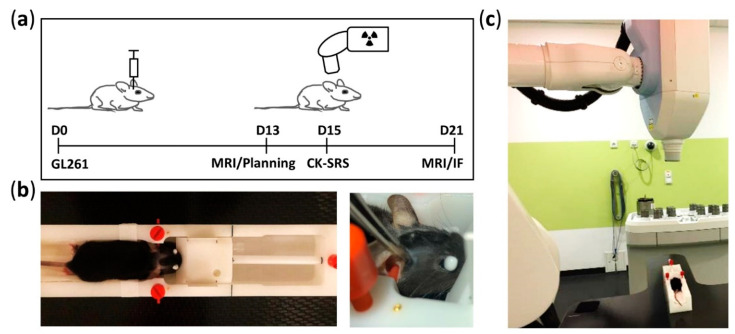
Timeline of experiment and details of mouse positioning during CK-SRS treatment. (**a**) Experimental timeline starting at day 0, defined as the date of the inoculation of GL261 glioma cells. At day 13, tumor growth was confirmed using MRI. Based on this acquired MRI dataset and the reference CT scan from one mouse, the treatment plan was prepared. Mice were subjected to CK-SRS treatment at day 15. At day 21, the final tumor volume was assessed by MRI and the mice were euthanized. (**b**) Manufactured MRI frame allowing three-point head fixation and equipped with gold fiducial markers. To ensure reproducible and accurate irradiation, the setting position of the fixation frame was marked (black lines) accordingly. (**c**) Representative image of animal positioning on the fixation frame placed on the treatment table of the CyberKnife system (Accuray Inc., Sunnyvale, CA, USA) during SRS.

GL261 cells were cultured in Dulbecco’s modified Eagle’s medium supplemented with 1% streptomycin/penicillin and 10% fetal bovine serum at 5% CO_2_ at 37 °C for 60–65 h before inoculation and were harvested at approximately 70% confluence. Anesthesia (9 mg ketamine-hydrochloride + 1 mg xylazine per 100 g bodyweight) was administered intraperitoneally before tumor cell inoculation, irradiation, and perfusion. On day 0, defined as the day of tumor inoculation (Figure 1A), each mouse received 10^5^ cells in 1 µL phosphate-buffered saline (PBS) via a stereotactic injection into the right striatum (2 mm lateral, 1 mm anterior, and 3 mm deep to the bregma) using a 1 µL Hamilton syringe. GL261 tumor-bearing animals without irradiation treatment were used as controls.

### 2.3. Fixation Frame

To ensure the reproducible positioning of mice during MRI and computed tomography (CT) scanning and irradiation treatment, we established a three-point head fixation frame made of polyoxymethylene (Figure 1B) and based on the MRI mouse bed (Mouse Tip SUC) from BRUKER (Billerica, MA, USA) equipped by a tooth bar and ear plugs. First, we modified the lower part of the frame for stable installation on the CK treatment table (Figure 1C). We then implanted four gold fiducial markers in the frame body to serve as reference points during CK-SRS. To ensure reproducible fixation for each mouse in the fixation frame, we lastly marked the positioning of the tooth bar and earplugs accordingly (Figure 1B).

### 2.4. Magnetic Resonance Imaging 

Mice were anesthetized via isoflurane inhalation (CP Pharma, approx. 1.2–1.4%) in 30% O_2_ and 70% N_2_O. MRI was conducted before irradiation and after 6 days using a 7 Tesla MRI scanner (BRUKER PharmaScan^®^, Billerica, MA, USA and Paravision 5.1 software) (Figure 1A). T1-weighted sequence using gadolinium contrast agent (Magnevist^®^, Bayer AG, Leverkusen, Germany) and T2-weighted sequence were used to analyze the tumor volume and radiation-induced edema (Analyze 10.0, AnalyzeDirect, Inc., Overland Park, KS, USA). Radiation-associated edema was calculated as the difference between T1- and T2-weighted volumes and is presented as a percentage of the total T1 volume as described previously [40].

### 2.5. CyberKnife Irradiation

After MRT examination on day 13 to verify tumor growth and prepare treatment plans, the animals received CK-SRS on day 15. MRI re-examination was performed to determine changes in tumor volume (Figure 1A). Furthermore, two animals were euthanized 1 h after irradiation to assess DNA double-strand breaks. Mice were under intraperitoneal narcosis during imaging and irradiation. 

To determine the basis for dose calculation and treatment tracking, one representative tumor-bearing mouse was placed in the fixation frame and assessed using a human CT scanner (Somatom Sensation Open, Siemens Healthcare, Erlangen, Germany). The voxel size was 0.15 × 0.15 × 0.60 mm³. This served as the reference CT dataset.

**Figure 2:**
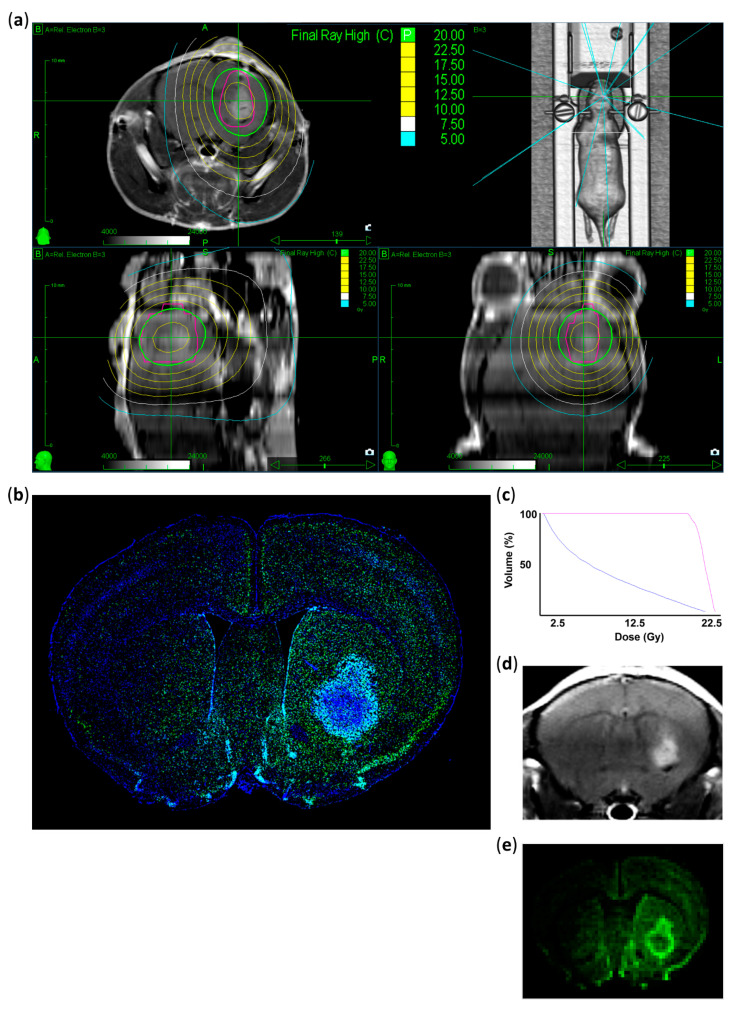
Treatment plan and histological validation of the irradiated area. (**a**) Representative MRI images showing the planned dose distribution in coronal, axial, and sagittal projections and the 3D reconstruction of planned beam delivery. The pink circle represents the PTV generated from the superimposed tumor volumes. Isodose lines in Gy with the prescribed dose are shown in green (20 Gy), ranging from 22.5 Gy (yellow) to 5 Gy (blue). (**b**) Representative immunostaining image indicating DNA double-strand breaks 1 h after CK-SRS treatment. Positively stained nuclei corresponding to the treatment plan are colored (γ-H2AX: green, DAPI: blue). (**c**) Dose–volume histogram of the irradiated tumor (pink) and normal mouse brain tissues (blue) indicating a rapid drop in dose. (**d**) Corresponding MRI image of the tumor immunostained in (**b**). (**e**) Representative image showing the summarized intensity voxels of γ-H2AX immunostaining, indicating that induced damage is the highest in the tumor and in the close peritumoral region corresponding to the PTV with decreasing intensity analogous to the isodose lines.

The MRIs of all mice irradiated in one session were co-registered with the planning CT data set (Precision 2.0, Accuray Inc., Sunnyvale, CA, USA). For each treatment session, a planning target volume (PTV) was generated from the superimposed tumor volumes delineated in the MRI scans. The contralateral brain was delineated to maintain a small dose as possible. The prescribed dose to the target was 1 × 20 Gy to 85%, resulting in a maximum dose of 23.5 Gy. By inversely optimizing the delivery of 6 MV photon beams at an approximate dose rate of 6.5 Gy/min and a diameter of 5 mm, an average target dose of 21.5 or 21.8 Gy (range: 19.4 or 18.6 Gy to 23.5 Gy) was achieved. The mean dose to the whole brain was 8 Gy (Figure 2A,C).

During irradiation, mice were positioned exactly in the three-point head fixation frame, similar to the CT and MRI set-up (Figure 1B,C) to ensure the automatic comparison of the fiducial (*n* = 4) positions in digital X-ray images reconstructed from CT with the current images acquired by CyberKnife (Accuray Inc., Sunnyvale, CA, USA) [1]. This alignment was performed on each mouse before irradiation. In total, 18/19 beams from multiple directions (Figure 2A) were delivered in a single session of approximately 8 min per mouse. The physical system accuracy was previously tested to be within 0.5 mm.

### 2.6. Immunofluorescent Staining

Anesthetized mice were intracardially perfused with 10 mL 0.9% sodium chloride followed by 4% paraformaldehyde (PFA) in PBS prior to brain harvesting. Two mice were euthanized 1 h after irradiation to assess induced DNA double-strand breaks, whereas the remaining animals were euthanized six days after irradiation (day 21). Mouse brains were fixed for 12–24 h in 4% PFA at 4 °C, followed by sucrose dehydration. After embedding in 2% gelatin, brains were cut into 10 µm sections using a microtome (Thermo Scientific (Microm HM 560), Waltham, MA, USA). DNA double-strand breaks were stained with rabbit anti-γ-H2A.X (Abcam (81299), Cambridge, UK) antibodies. After drying at 20 ± 1 °C, sections were incubated for 30 min in 0.1% Triton in PBS prior to 30 min incubation blocking with the blocking solution (1% BSA, 22.52 mg) in PBST (PBS + 0.1% Tween 20) or donkey serum (10% in PBS). Primary antibodies (anti-γ-H2A.X) were used at 1:200 dilution and incubated for 60 min. Sections were washed in 0.2% Tween 20 in PBS and incubated with secondary antibodies (Alexa Fluor^®^ 488 donkey anti-rabbit, Jackson ImmunoResearch (711-545-152), West Grove, PA, USA, 1:200) for 1 h. The entire protocol was performed at 20 ± 1 °C. Antibodies were dissolved in 1% BSA and 0.1% Tween 20 in PBS.

The perfusion protocol for proliferation and apoptosis staining slightly differed as intracardial perfusion was performed with 4% PFA only, and brains were fixed for 24 h in 4% PFA at 4 °C before saccharose dehydration. For proliferation (rabbit anti-Ki67, Thermo Fisher Scientific (RM-9106-S1), Waltham, MA, USA, 1:200) and apoptosis (TUNEL, ApopTag red In situ kit (S7165), Merck Millipore, Darmstadt, Germany) staining, we performed a combined protocol. First, we performed apoptosis staining according to the manufacturer’s protocol. Second, Ki67 primary antibodies were incubated for 16 h (4 °C) after permeabilization with Triton 0.3% in PBS and 1 h blocking with 1% casein in PBS. Prior to incubation with the secondary antibodies (Alexa Fluor^®^ 647 donkey anti-rabbit, Jackson ImmunoResearch (711-605-152), West Grove, PA, USA, 1:200) for 120 min at 20 ± 1 °C, sections were washed with Casein 0.5% in PBS. Tumor-associated microglia and macrophages (TAMs) (goat anti-Iba1, Abcam, (ab5076), Cambridge, UK, 1:200) and vessels (rat anti-CD31, BD Pharmingen (550274), San Diego, CA, USA, 1:50) were stained. An autofluorescence eliminator reagent (Merck Millipore (2160)) was used according to the manufacturer’s protocol. Then, the slices were washed and blocked with casein 1% casein in PBS for 30 min, prior to incubation with primary antibodies for 2 h at 20 ± 1 °C. Slices were washed with casein 0.5% in PBS and incubated with secondary antibodies at 90 min and 20 ± 1 °C (Alexa Fluor^®^ 647 donkey anti-goat, Jackson ImmunoResearch (705-605-147), 1:200 and Cy^TM^3 donkey anti-rat, Jackson ImmunoResearch (712-165-153), 1:200). All antibodies were dissolved in 0.5% casein in PBS. Nuclei were stained with DAPI (Mounting Media, Dianova, Hamburg, Germany) after washing with PBS and distilled water. Images were obtained using a fluorescence microscope (Zeiss, Axio Observer Z1, Zeiss MicroImaging GmbH, Jena, Germany) and analyzed using ImageJ (1.52p, NIH, Bethesda, MD, USA).

### 2.7. Statistics

Data are shown as the mean ± standard deviation calculated from the individual values of the animals in the respective groups. Independent Student’s *t*-tests were performed to determine the differences between the control and irradiated groups. Statistical significance was set at *p* ≤ 0.05. All analyses were performed using GraphPad Prism (v 6.01, GraphPad Software, San Diego, CA, USA).

## 3. Results

### 3.1. Verification of the Irradiated Area

Radiation therapy effectiveness mainly depends on radiation-induced DNA damage, leading to cell cycle arrest and cell death [41]. γ-H2AX accumulates in a dose-dependent manner after the formation of DNA double-strand breaks and is therefore a good marker for DNA damage [42,43,44]. Thus, we performed γ-H2AX staining to verify the localization of the delivered radiation. Brain tissues collected 1 h after irradiation showed γ-H2AX enrichment (DNA double-strand breaks) exactly at the planned target dose delivery region (Figure 2A,B,D,E). Decreased intensity of γ-H2AX-positive signals was observed in the middle of the contralateral hemisphere, which matched the previously planned irradiation volume. Highly γ-H2AX positive cells were mainly found within the vital tumor areas and their direct surroundings, which also coincided with our planned volume as revealed by the comparison of planning target volume (PTV) and γ-H2AX immunostaining patterns (Figure 2A,B). Weakly γ-H2AX-positive cells were also detected in the contralateral hemisphere, especially in the cortical areas (Figure 2B).

To quantify the difference between irradiated and non-irradiated areas, we measured γ-H2AX intensity in the defined regions of interest (Figure S1C). In the contralateral hemisphere, only 4–6% of the intensity of the irradiated tumor was measurable. In contrast with the clearly detectable DNA double-strand breaks 1 h after irradiation, no enrichment of γ-H2AX positive nuclei was found in the irradiated area after six days (Figure S1A,B).

### 3.2. Assessment of Tumor Growth and Edema Formation

**Figure 3:**
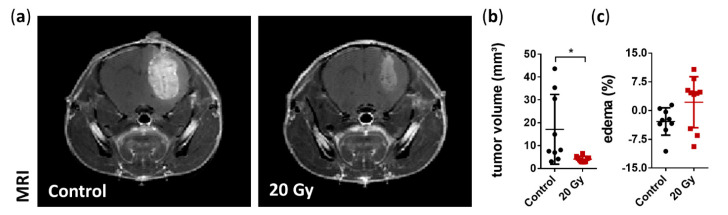
MRI assessment of the tumor volume and peritumoral edema at day 21. (**a**) Representative coronal MRI T1-weighted images with contrast agent highlighting the smaller tumor following irradiation. (**b**) Volumetric analysis demonstrated a significant reduction in tumor volume six days after irradiation treatment compared to non-irradiated tumors (data are presented as the mean ± SD of each group. Individual values of each animal are provided as a single dot; *n* = 9–10 per group; * *p* < 0.05). (**c**) Quantification of edema size showed no significant changes; however, a large variability within the irradiated group was observed. Edema was calculated as the difference in the tumor volume between the T2- and T1-weighted MRI images and presented as the percentage of total tumor volume (data are presented as the mean ± SD of each group. Individual values of each animal are provided as a single dot; *n* = 9–10 per group).

The effect of irradiation on tumor growth was assessed as an indirect indicator of radiotherapy precision. Irradiation with 20 Gy in a single fraction significantly reduced the tumor volume by 76.3% six days after treatment (irradiated: 4.06 ± 1.27 mm³ vs. non-irradiated: 17.15 ± 15.27 mm³ on day 21; *p* = 0.0149; Figure 3A,B). Additionally, we assessed the possible alterations in the common peritumoral edema on MRI as an indicator of radiation-induced higher permeability of the tumor vasculature compared to non-irradiated tumors [45,46]. We found that CK-SRS treatment showed no significant changes in edema in irradiated tumors; however, a large variability within the irradiated group was observed (irradiated 2.2% ± 6.67% vs. non-irradiated −2.84% ± 3.58%; *p* = 0.598; Figure 3C).

### 3.3. Assessment of the Early Effects of Irradiation Therapy

**Figure 4:**
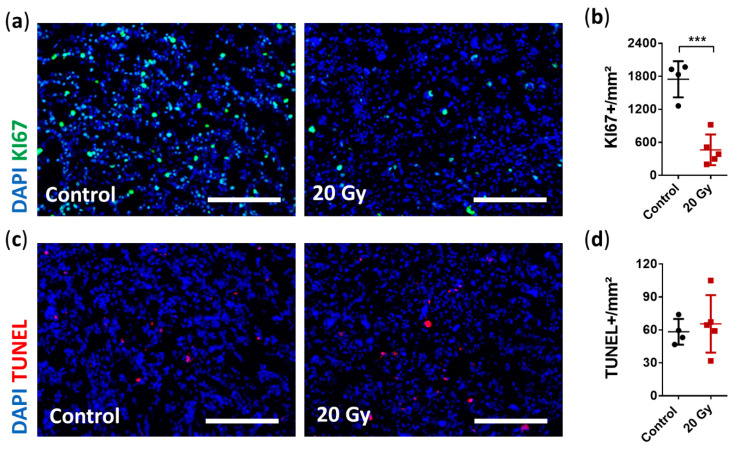
Immunohistological analysis of early radiobiological effects on cellular proliferation and apoptosis. (**a**) Representative immunostaining images showing severely impaired proliferation activity after irradiation (Ki67: green, DAPI: blue; scale bar = 200 µm). (**b**) Quantification of cellular proliferation demonstrated a decrease by 79% in irradiated tumors compared to non-irradiated tumors (data are presented as the mean ± SD of each group. Individual values of each animal are provided as a single dot; *n* = 4–5 per group; *** *p* < 0.001). (**c**) Representative immunostaining images showing no change in cell apoptosis (TUNEL: red, DAPI: blue; scale bar = 200 µm). (**d**) Quantification of apoptotic cells showed similar amounts of TUNEL-positive cells per mm² between the irradiated and non-irradiated tumors (data are presented as the mean ± SD of each group. Individual values of each animal are provided as a single dot; *n* = 4–5 per group).

Irradiation-induced DNA damage disrupts the proliferation cycle and in case of irreparability leads to death or senescence of the affected cells [47]. To analyze the early effects of SRS, we first assessed cell proliferation using Ki67 and apoptosis using TUNEL staining. Immunofluorescence analysis revealed a significant decrease of 79% in cell proliferation (Ki67^+^: irradiated 464.0 ± 279.6 cells/mm² vs. non-irradiated 1748.0 ± 328.0 cells/mm²; *p* = 0.0004; Figure 4A,B). In contrast, apoptosis was not increased based on the short-term observation period of 6 days following SRS treatment (TUNEL^+^: irradiated 65.5 ± 26.2 cells/mm² vs. non-irradiated 58.4 ± 11.7 cells/mm²; *p* = 0.6311; Figure 4C,D).

**Figure 5:**
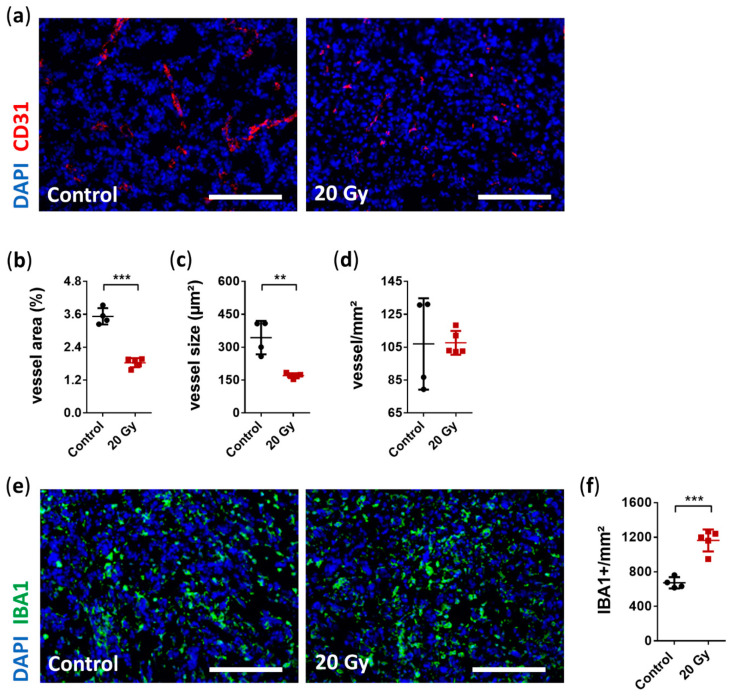
Immunohistological analysis of early radiobiological effects on tumor vasculature and the accumulation of tumor-associated microglia and macrophages (TAMs). (**a**) Representative immunostaining images of reduced tumor vasculature after irradiation (CD31: red, DAPI: blue; scale bar = 200 µm). (**b**) Quantitative analysis revealed a significantly decreased total vessel area in the irradiated group compared with the non-irradiated group (data are presented as the mean ± SD of each group. Individual values of each animal are provided as a single dot; *n* = 4–5 per group; *** *p* < 0.001). (**c**) Average vessel size was also significantly reduced after irradiation (data are presented as the mean ± SD of each group. Individual values of each animal are provided as a single dot; *n* = 4–5 per group; ** *p* < 0.01). (**d**) In contrast, there was no difference in vessel counts per mm² between the irradiated and non-irradiated group (data are presented as the mean ± SD of each group. Individual values of each animal are provided as a single dot; *n* = 4–5 per group). (**e**) Representative immunostaining images showing increased TAM accumulation six days after irradiation (Iba1: green, DAPI: blue; scale bar = 200 µm). (**f**) Quantification of positively stained Iba1 cells per mm² showed enhanced accumulation of 73% in irradiated vs. non-irradiated group (data are presented as the mean ± SD of each group. Individual values of each animal are provided as a single dot; *n* = 4–5 per group; *** *p* < 0.001).

A well-known characteristic of GBM biology is pronounced angiogenesis [48]. Thus, the destruction of the tumor vasculature with the subsequent deprivation of oxygen and nutrients in the irradiated area is an important mechanism for the success of irradiation therapy [45]. Therefore, we focused on the tumor vasculature in this study. Structural changes in the tumor vasculature, including significantly reduced vessel area and average vessel size, were observed; however, vessel density was unaffected (Figure 5A–D). Vessel area and average vessel size were significantly reduced by approximately 48% and 50%, respectively (vessel area relative to the total area: irradiated 1.84% ± 0.17% vs. non-irradiated 3.53% ± 0.30%, *p* < 0.0001, Figure 5A,B; average vessel size: irradiated 170.5 ± 11.33 µm² vs. non-irradiated 344 ± 76.29 µm², *p* = 0.0014, Figure 5A,C; and vessel density: irradiated 107.7 ± 7.3 vessel/mm² vs. non-irradiated 107 ± 27.8 vessel/mm², *p* = 0.96, Figure 5A,D).

Neuroinflammation induced by irradiation is accompanied by the accumulation of microglia [18,49,50]. Resident microglia are a source of proangiogenic factors supporting tumor angiogenesis, thus highlighting the crucial role of resident microglia in pathogenesis of GBM [51]. We assessed whether irradiation induced any short-term alteration in TAM accumulation. Interestingly, we observed a strong immune response 6 days after irradiation with a significant increase in TAM accumulation of 73% (Iba1_+_: irradiated 1163.0 ± 127.2 cells/mm² vs. non-irradiated 672.2 ± 65.3 cells/mm², *p* = 0.0002, Figure 5E,F).

## 4. Discussion

In this study, we successfully administered CK-SRS treatment on a syngeneic tumor-bearing mouse model using a single reference CT and the superimposed tumor volumes, thereby optimizing the irradiation process. Analysis of the radiation delivery using γ-H2AX staining confirmed radiation precision as evidenced by DNA damage within GBM-infiltrated brain regions and their surroundings while preventing damage in the normal mouse brain tissues. Reduction in tumor growth and decreased cell proliferative activity also indicated successful tumor irradiation. Furthermore, we detected alterations in tumor vascularization and TAM accumulation as early effects of single high dose CK-SRS treatment. 

The radiobiology of whole-brain irradiation was investigated in 1982, focusing on the long-term effects on vascularization [16]; however, the intracranial radiobiological effects of SRS remain poorly understood [41,52,53,54]. Thus, in this study, we focused on the establishment of an experimental mouse model for CK-SRS treatment, which could serve as a foundational basis for further studies. On one hand, the availability of specific small radiation platforms (i.e., SmART and SARRP) for animals is limited; on the other hand, the beam quality is different among these platforms, with higher energies for human irradiation platforms (approximately 200 kV vs. 6 MV). We are convinced that the application of a human irradiation device with clinical beam quality, dose rate, and dose in experimental setups provides the most realistic results on the radiobiological effects of SRS. The available experimental studies on healthy mouse brains are summarized in Table 1. Notably, only a few groups have examined the irradiation of brain tumors in mice, none of which utilized CyberKnife [12,14,36,37,38,39]. Therefore, to the best of our knowledge, our study represents the first brain tumor irradiation of mice using CyberKnife.

The mouse frame and the irradiation protocol particularly developed for our mouse model allow accurate positioning without invasiveness. Other studies used either body molds with no fixation points for the attachment of the mouse head or glue to fixate collimators on mouse skin [14,33,37]. However, these approaches lack of reproducibility and accuracy. To achieve high and reproducible accuracy, we used a three-point fixation system equipped with four gold fiducial markers that were integrated into the frame; therefore, invasive reference markings were not required. Because of the precise planning in our study, only the tumor and peritumoral areas were subjected to irradiation, and half of the prescribed dose covered a brain volume of approximately 35%. This is of major importance for the comparative assessment of the effects of SRS on healthy tissues surrounding the tumor and non-irradiated tissues. Remarkably, the minimum collimator diameter of 5 mm using our CyberKnife system was within the range of specialized irradiation platforms for small animals whose size ranges between 3 × 3 mm and 5 × 5 mm [12,13,14].

In case of intracranial tumor irradiation, Yahyanejad et al. used two opposing beams with a diameter of 3 or 5 mm depending on the tumor size [12], whereas Baumann et al. delivered irradiation using a single beam superior-to-inferior after determination of isocenter depth within an area of 5 × 5 mm [14]. Although the dose gradient was steeper in these treatments, the beam superposition of our treatment allows for a highly conformal dose distribution in the tumor, which is similar to clinical conditions. Thus, the volume receiving 20 Gy was estimated to be only 6% of the whole brain parenchyma, which was comparable to previous studies using small animal devices [35,36,37]. The only published irradiation study of a mouse brain using CyberKnife by Kim et al. demonstrated its ability to successfully and precisely irradiate an entire hemisphere [33]. Compared to this study, we preserved more of the normal mouse brain brain tissue with a shorter treatment time [33]. In addition, the reduction in tumor volume and cell proliferation after SRS treatment compared to untreated tumors indicated the effectiveness and precision of the irradiation using only one reference CT data set for all tumors and an overlaid target volume from each tumor MRI scan. This is especially important in terms of reducing and refining strategies in animal experiments as only one animal had to undergo additional anesthetization for the CT scan in our study.

To irradiate as little healthy tissue as possible and to use only one reference CT data set during planning, tumors should be implanted in a defined location. Here, we assessed the correct inoculation without major inter-individual differences two weeks after implantation using MRI. The prescribed dose of 20 Gy was adapted to the clinical routine as we used 20–21 Gy as a single dose for brain metastases and recurrent small-volume GBM. This was significantly less than the published lethal dose (LD_50_) of 32.4 Gy within 300 observation days in C3Hf/Sed/Kam mice [17]. Particularly, for the C57BL/6 strain, doses up to 35 Gy in a single fraction on the healthy brain only resulted in weight loss without causing higher mortality within 12 months [50]. Furthermore, Balb/c mice were irradiated with up to 60 Gy in a single fraction in one hemisphere, suggesting that any adverse effects would be absent at a much higher dose over an observation period of several months [34]. Yahyanejad et al. employed 12 Gy to irradiate U87MG tumors in CD1 nu/nu mice and reported that this treatment prolonged survival compared to lower doses [12]. These findings indicate that irradiation can be safely applied at higher doses for the treatment of intracranial tumors. Therefore, we speculated that a dose of 20 Gy was safe in regard to dose-induced severe side effects. In this study, none of the irradiated animals exhibited harmful side effects such as development of new neurological deficits or loss of weight and survived during the observation period. However, we detected circular hair loss in the skin, which was partly in the trajectory of the main beam path directly above the tumor. Although hair loss is a common side effect of radiation and can be tolerated, we have also observed this phenomenon in non-irradiated animals. Therefore, we attribute this to scarring following tumor inoculation.

TAMs are of great interest in terms of therapeutic efficacy because they create an immunosuppressive microenvironment in both brain metastases and GBM [56,57,58]. In this study, we detected a markedly enhanced accumulation of TAMs despite the significantly reduced tumor volume early after irradiation. The accumulation of TAMs within the tumor and its surroundings correlates with the tumor histological grade and thus promotes tumor growth [59]. Therefore, we attribute this high TAM accumulation in the early phase to a neuroinflammatory reaction [18,49,50]. The immunomodulatory effect of irradiation is also a major aspect of how immune cells interact with cancer cells within the tumor microenvironment in response to therapy [60,61,62]. Thus, changes in the TAM accumulation should be verified in the future in more detail. Interestingly, Riva et al. observed that at a lower dose of 4 Gy delivered in a single fraction, irradiation resulted in the reduction of not only the total number of TAMs in the short term, but also the fraction of TAMs with the immunosuppressive M2-phenotype, which promotes a protumorigenic environment [15]. This underscores the need for an experimental protocol using similar dose and fractionation to that used for SRS treatment in humans to verify the underlying mechanisms in clinical response. In view of the early changes in the tumor vasculature with significantly reduced total vessel area and average vessel size in the irradiated group compared to the non-irradiated group, more in-depth investigations such as alterations in desmin coverage and angiogenic pathways including VEGF and CXCR2 are justified in future experiments with longer observation period.

To establish CK-SRS treatment in an intracranial tumor mouse model, a well-established experimental GBM brain tumor model using GL261 murine cells from immune-competent mice was selected [63]. After in vivo inoculation, the GL261 tumor cell line showed relatively rapid and aggressive tumor growth in a homogeneous tumor cell population but only moderate invasive growth [63,64]. This growth pattern is therefore comparable to brain metastases in terms of its low invasiveness, as demonstrated in the supplementary figure (Figure S1D). Overall, the GL261 GBM tumor model was qualified for the establishment of CK-SRS and provided new insights on the direct early effects of SRS on the tumor vasculature and the reactivity of the immune system. However, the rapid growth of the non-treated GL261 tumors limits the observation period to around 21 days after tumor inoculation, thus 6 days after the CK-SRS was chosen to provide a comparison of irradiated to non-irradiated tumor tissue. For the assessment of late irradiation effects less aggressive tumor cell lines may be favorable. In this context, it should also be emphasized that GBM is not the most common oncological indication for radiosurgery, thus we are convinced that our proposed model with the described irradiation protocol using CK-SRS should be applied to other brain tumors. As brain metastases are the main indication for SRS treatment in routine clinical practice, the next step is to conduct these experiments using the most common metastatic cell lines.

In this study, we had to overcome several pitfalls as we utilized a human SRS setup to treat mice. Planning CT scanning was performed using thin tissue slices; however, no dedicated small animal CT scanner was available, which may lead to inaccuracies in image fusion and planning. Nevertheless, DNA double-strand breaks were detected within the irradiation region, thereby confirming the ability to target the correct tumor volume. Furthermore, there was no suitable standardized frame available for purchase. Therefore, for this study, the mouse frame with a non-invasive three-point head fixation and integrated with gold fiducial markers was custom-made in advance. However, its fabrication is only necessary once as it can be repeatedly used. Moreover, we did not track the mouse brain itself using the CyberKnife, but only focused on the fiducial markers in the fixation frame. To account for this, the X-ray fusion of each mouse skull was visually checked for correct positioning. Importantly, the selected size of the collimator was the upper limit for such small volumes. However, this limitation can be addressed using multiple beams from different directions. Finally, it should be pointed out that for a real understanding of radiation biology after radiosurgical treatment, different tumors with different proliferation rates and a detailed assessment of the therapeutic effects at different points in time are necessary. Here we have only presented the data from a single cell line, so future experiments with this established radiation model are warranted, especially with common metastatic cell lines and, more preferably, with patient-related cell lines as well.

## 5. Conclusions

Our study established a murine model and experimental protocol for the precise and effective application of CK-SRS treatment for orthotopic GBM, thus allowing the direct targeting of the tumor volume while protecting most of the normal mouse brain tissues. The use of clinical megavolt beams for small animal studies is promising as irradiation using CK can be delivered from multiple beam directions, thereby reducing directional dose dependence, including build-up effects. The established workflow enabled the easy handling of mice during the experiments and did not require invasiveness for accurate positioning; thus, animals were subjected to reduced stress. Our established model provides a foundation basis for future studies focusing on the effects of intracranial irradiation. Because dedicated small animal radiation machines are rare compared to linear accelerators for humans and must be purchased separately, CK enables small animal studies alongside routine clinical patient care.

## Figures and Tables

**Table 1:** Mouse models used for intracranial irradiation studies.

Author [Reference]	Year	Irradiation Equipment and Dose Rate (Gy/min)	Dose (Gy)	Target	Observation Period	Focus of Investigation
Yoshii Y., et al. [16]	1982	250 kVp X-rays at 120 rads/min	13–25 Gy	Whole brain	177 weeks	Fibrinoid necrosis and tissue damage
Chiang C.S., et al. [17]	1993	Phillips orthovoltage machine (250 kV, 15 mA, 2,38 Gy/min)	2–45 Gy	1 × 0.5 cm of left hemisphere	360 days	Neurological changes and changes in myelination
Chiang C.S., et al. [18]	1993	Phillips orthovoltage machine (250 kV, 15 mA, 2.38 Gy/min)	2–45 Gy	1 × 0.5 cm of left hemisphere	360 days	Response of astrocytes and microglia
Hong J.H., et al. [19]	1995	Phillips orthovoltage machine (250 kVp, 15 mA, 2.38 Gy/min)	25 Gy	Midbrain (1 × 0.5 cm left lateral field)	48 h	In vivo molecular response to Rtx
Chow B.M., et al. [20]	2000	Two Picker Gemini 100 kV X-rays units	2 Gy	Whole brain	36 h	Role of p53 in irradiation induced apoptosis in adult CNS
Daigle J.L., et al. [21]	2001	Phillips orthovoltage X-ray system (250 kVp, 2.88 Gy/min)	25 Gy	Midbrain	9 months	Role of TNF-α in irradiation induced apoptosis in adult CNS
Gaber M.W., et al. [22]	2003	6 MV linear accelerator (3.5 cm beam, 3 Gy/min)	10, 20, 40 Gy (fractionated/single)	Whole brain	8 days	Molecular response, rt-PCR (ICAM-1, TNF-α)
Mizumatsu S., et al. [23]	2003	Phillips orthovoltageX-ray system (1.75 Gy/min, 5 × 6 cm treatment field)	1, 2, 5, 10 Gy	Whole brain	48 h	Neurogenesis after Rtx (apoptosis, proliferation, immature neurons)
Rola R., et al. [24]	2004	Phillips orthovoltage X-ray system (1.75 Gy/min)	2, 5, 10 Gy	Whole brain	3 months	Dose response of proliferating SGZ cells, Neuroinflammation/ neurogenesis
Yuan H., et al. [55]	2006	Siemens 6-MV X-ray linear accelerator	40 Gy in total (2 Gy/day, 5 days/week, 4 weeks)	4 × 6 mm cranial window	6 months	Acute and long-term effects of fractionated Rtx on BBB permeability
Ansari R., et al. [31]	2007	Siemens 6-MV X-ray linear accelerator (3.5 cm collimator, 3 Gy/min)	20 Gy	Whole brain/single hemisphere	4 months	Effects of Rtx on brain vasculature
Jost S.C., et al. [32]	2009	micro-RT system	60 Gy in total (10 × 6 Gy over 2 weeks)	Left hemisphere	4 months	Determination of radiation-induced necrosis using MRT
Mao X.W., et al. [25]	2010	600 MeV/nucleon ^56^Fe particles (18 × 18 cm beam, 1.5–2.5 Gy/min)	0.5, 2, 4 Gy	Whole Brain	12 months	Irradiation-induced changes in microvessel endothelial population from dentate gyrus/cornu ammonis
Wu K.L., et al. [26]	2010	2 × 160 kV X-ray beams	up to 35 Gy	Whole brain	8 months	Role of ICAM-1 in the pathogenesis of brain injury after Rtx
Ford E.C., et al. [13]	2011	Small Animal Radiation Research Platform (SARRP)	10 Gy	Lateral ventricle wall, dentate gyrus of hippo- campus	4 weeks	Validation of Rtx accuracy (γH2AX); Proliferation (Ki67)
Moravan J.M., et al. [50]	2011	^137^Cs irradiator (1.25 Gy/min)	0–30 Gy	0.5 × 12.5 cm window	12 months	Neuroinflammation after Rtx
Rao A.A., et al. [27]	2011	^137^Cs lateral beam	20 Gy in total (4 Gy/day for 5 days)	Whole brain	1 month	Effect of fractionated irradiation on hippocampal function
Belarbi K., et al. [28]	2013	^137^Cs collimator	10 Gy	Whole brain	82 days	Role of CCR2 in the pathogenesis of irradiation injury to hippocampal neurons
Kim H., et al. [33]	2014	CyberKnife	3 Gy	Single hemisphere	20 min	Validation of Rtx accuracy (γH2AX)
Son Y., et al. [29]	2014	6 MV high energy photon rays (ELEKTA, 3.8 Gy/min)	1, 10 Gy	Whole brain	3 months	Late irradiation-induced effects on hippocampal function
Morganti J.M., et al. [30]	2014	Phillips orthovoltage X-ray system	10 Gy in total (5 Gy/hemisphere)	Whole brain	28 days	Resident (CX3CR1+) vs peripheral (CCR2+) innate immune response in the brain following cranial irradiation
Jiang X., et al. [34]	2015	Gamma Knife	45, 50, 60 Gy (single fraction); 60 Gy (3 fractions every 2 days	Single hemisphere	22 weeks	Radiation necrosis (H&E, PTAH)
Zarghami N., et al. [35]	2015	microCT/RT system	16 Gy	Single hemisphere	30 min	Validation of Rtx accuracy (γH2AX)
**WITH TUMORS**						
Baumann B.C., et al. [14]	2012	SARRP	20 Gy	U251 glioblastoma	60 min	Validation of Rtx accuracy (γH2AX)
Perez-Torres C.J., et al. [39]	2014	Gamma Knife	50 Gy (healthy brain) 3 × 7.5 Gy (glioma)	Healthy brain DBT glioma	Survival study	Differentiation tumor vs. irradiation necrosis (DWI MRI)
Yahyanejad S., et al. [12]	2015	Photon spectrum of 225 kVp at 12 mA (3 Gy/min)	4, 8, 12 Gy	Glioblastoma (U87MG)	Survival study	In vivo test of Rtx accuracy
Hartmann J., et al. [37]	2016	LINAC	10 × 1.8 Gy	Craniopharyn- gioma (0.6 × 0.7 × 0.5 cm)	60 min after 2 or 3 fractions	Validation of Rtx accuracy (γH2AX)
Miki S., et al. [38]	2018	CP-160 (Acrobio, X-rays, 0.33 Gy/min)	3 × 4 Gy	Glioma (U87MG)	Survival Study	Changes in vascularization and tumor volume
Zarghami N., et al. [36]	2018	Micro-CT/RT system	8, 12, 24 Gy	Half brain (breast metastasis)	11 days	Validation of Rtx accuracy (γH2AX)

## Data Availability

The data presented in this study are available on request from the corresponding author. The data are not publicly available due to the protection of data privacy.

## Supplementary Materials

The following are available online at https://www.mdpi.com/article/10.3390/cancers13143416/s1, Figure S1: Demonstration of γ-H2AX staining and tumor growth characteristics of GL261.
