# Supplementary material for: Establishment and Validation of CyberKnife Irradiation in a Syngeneic Glioblastoma Mouse Model
Source: Cancers (Basel). 2021 Jul 8;13(14):3416. doi: 10.3390/cancers13143416 (PMC8303959; doi:10.3390/cancers13143416)
Supplement: Supplementary file 1 [file cancers-13-03416-s001.zip › cancers-1241072-supplementary.pdf]

cancers

MDPI

Supplementary Materials: Establishment and Validation of CyberKnife Irradiation in a Syngeneic Glioblastoma Mouse Model

Claudius Jelgersma, Carolin Senger, Anne Kathrin Kluge, Anastasia Janas, Melina Nieminen-Kelhä, Irina Kremenetskaia, Susanne Mueller, Susan Brandenburg, Franziska Loebel, Ingeborg Tinhofer, Alfredo Conti, Volker Budach, Peter Vajkoczy and Gueliz Acker

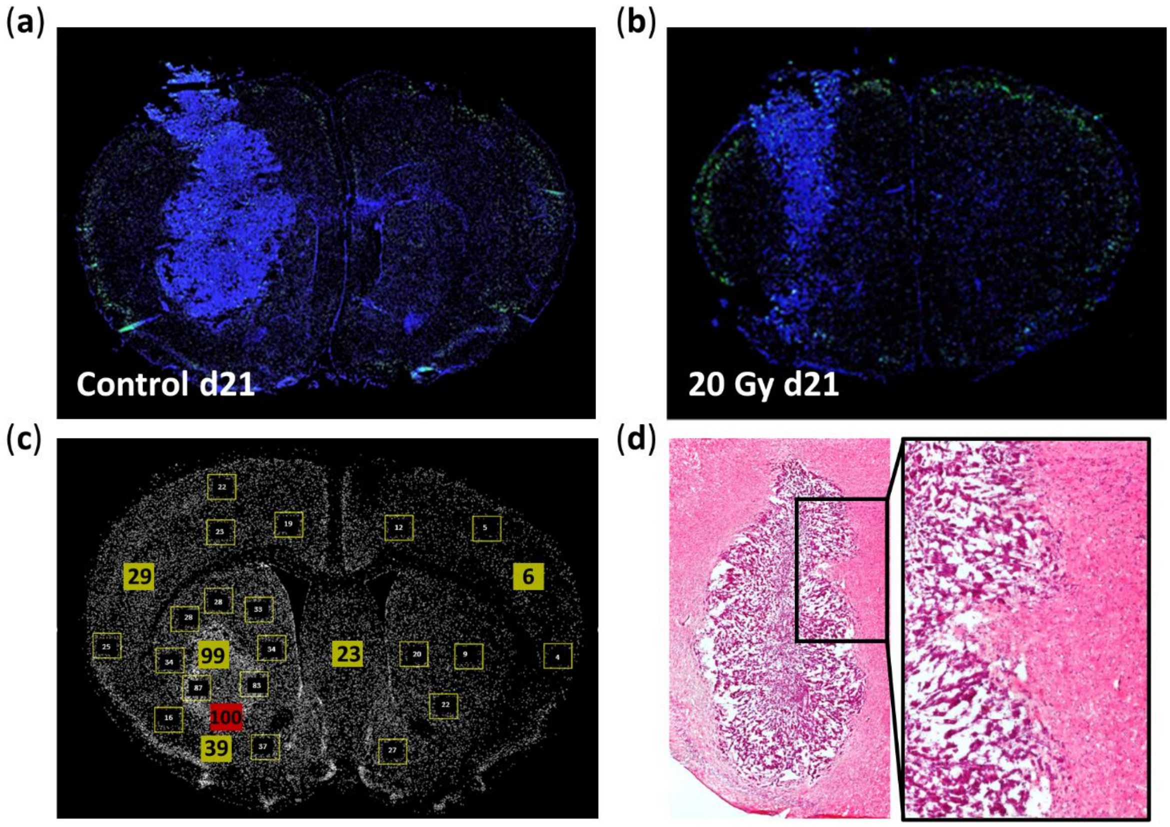

Figure S1. Demonstration of γ-H2AX staining and tumor growth characteristics of GL261. (**a**) Representative γ-H2AX immunostaining image of the non-irradiated tumor (control) on day 21. Unspecific γ-H2AX staining is observed in the cortical and subcortical regions (γ-H2AX: green, DAPI: blue). (**b**) Representative γ-H2AX immunostaining image on day 21 showing no specific DNA damage in the irradiated area 6 days after irradiation (γ-H2AX: green, DAPI: blue). (**c**) γ-H2AX intensity was measured 1 h after irradiation in the defined regions of interest (yellow rectangle) and expressed as percentage of the reference intensity (red rectangle). (**d**) Representative HE staining image of the non-irradiated tumor on day 21 showing the non-infiltrative character of GL261 tumors.
